# Progress on highly proton-conductive polymer thin films with organized structure and molecularly oriented structure

**DOI:** 10.1080/14686996.2020.1722740

**Published:** 2020-02-14

**Authors:** Yuki Nagao

**Affiliations:** School of Materials Science, Japan Advanced Institute of Science and Technology, Nomi, Japan

**Keywords:** Interface, substrate dependence, thickness dependence, molecular weight dependence, molecular ordering, fuel cells, 101 Self-assembly / Self-organized materials, 212 Surface and interfaces, 207 Fuel cells / Batteries / Super capacitors, 206 Energy conversion / transport / storage / recovery, 306 Thin film / Coatings, 504 X-ray / Neutron diffraction and scattering, 505 Optical / Molecular spectroscopy

## Abstract

Several current topics are introduced in this review, with particular attention to highly proton-conductive polymer thin films with organized structure and molecularly oriented structure. Organized structure and molecularly oriented structure are anticipated as more promising approaches than conventional less-molecular-ordered structure to elucidate mechanisms of high proton conduction and control proton conduction. This review introduces related polymer materials and molecular design using lyotropic liquid crystals and hydrogen bond networks for high proton conduction. It also outlines the use of substrate surfaces and external fields, such as pressure and centrifugal force, for organizing structures and molecularly oriented structures.

## GRAPHICAL ABSTRACT

**Figure:**
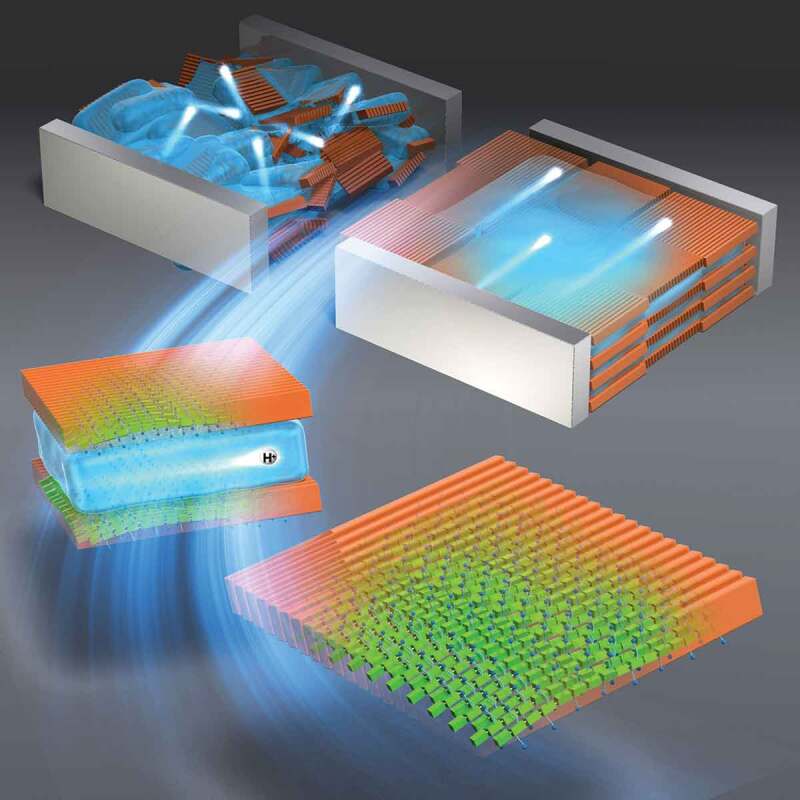

## 1. Introduction

Since the usefulness of proton-conductive polymer membranes in fuel cells was demonstrated by Grubb at General Electric in the 1950s [1,2], proton-conductive polymers have come to be used not only for ion exchange membranes but also for fuel cell membranes. After two oil shocks in the 1970s and development through space programs, fuel cells became known to society as an alternative energy source with high energy conversion efficiency. The design of proton-conductive polymers has long been based on phase segregation between a hydrophobic matrix and hydrophilic channels [3–9]. Most proton-conductive polymers did not have a long-range molecular order. It was only possible to elucidate the domain size of the phase-segregated structure using small and wide-angle X-ray scattering, atomic force microscopy, and transmission electron microscopy. Discussing the correlation between structure and proton conduction more deeply was not easy.

**Figure 1.**
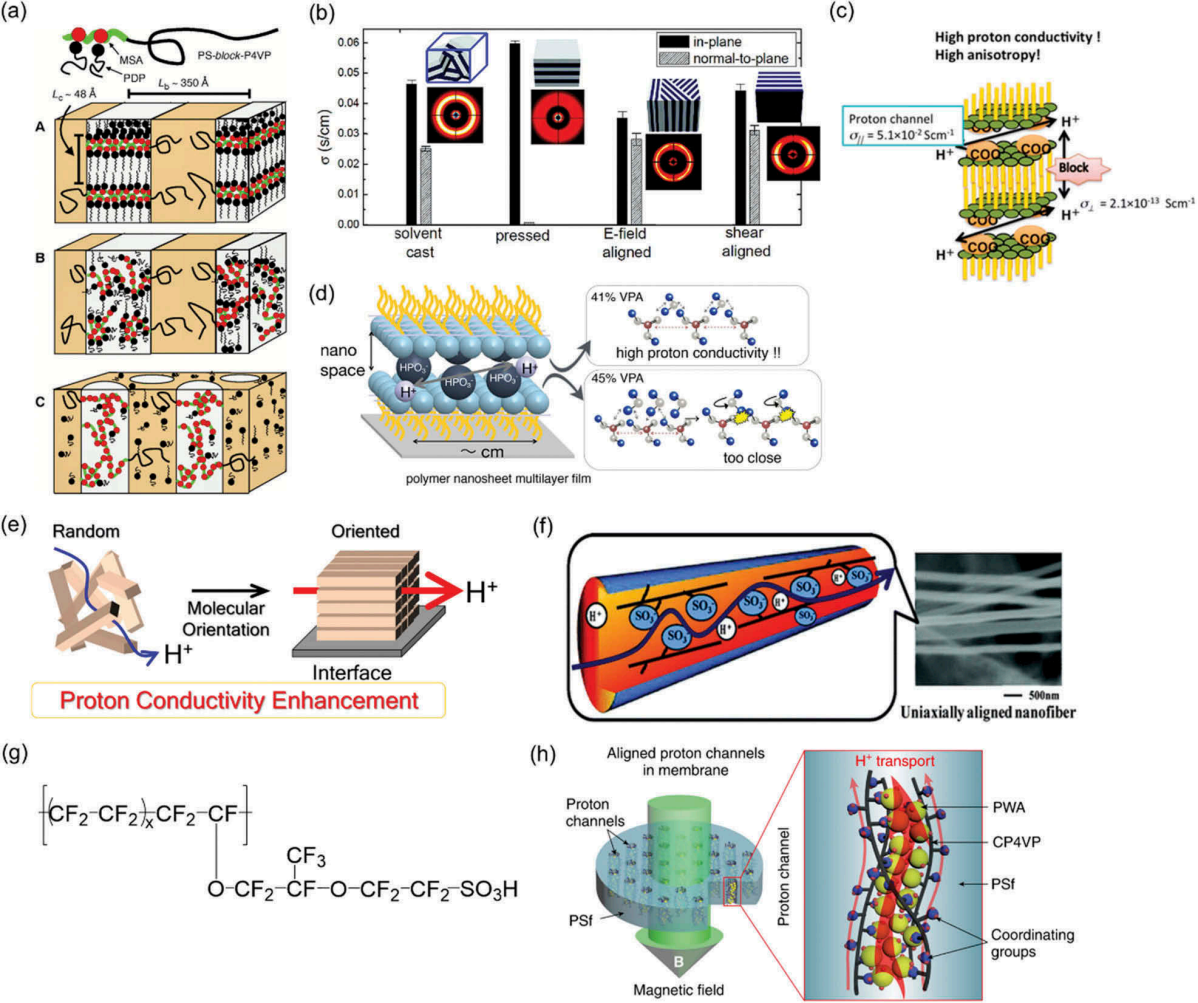
Schematic of proton-conductive polymer films using an organized structure and oriented structure. (a) Schematic of the self-organized structures of PS-*block*-P4VP(MSA)_1.0_(PDP)_1.0_. The local structures are indicated; macroscopically, the samples are isotropic. Reprinted with permission from Ruokolainen et al. [10]. Copyright 1998. The American Association for the Advancement of Science. (b) 2D small angle X-ray scattering profiles and in-plane and normal-to-plane conductivity of as-cast and aligned samples. Reprinted with permission from Park et al. [11]. Copyright 2009 American Chemical Society. (c) Schematic of multilayer film of poly(*N*-dodecylacrylamide-*co*-acrylic acid) by Langmuir–Blodgett method with highly anisotropic proton conduction. Reprinted with permission from Sato et al. [13]. Copyright 2015 American Chemical Society. (d) Model of distance of each acidic group for high proton conduction. Reprinted with permission from Tsukamoto et al. [16]. Copyright 2019 American Chemical Society. (e) Schematic of proton conduction enhancement by molecular orientation of proton-conductive polymers. (f) Schematic of aligned electrospun nanofiber of sulfonated polyimide. Reprinted with permission from Tamura et al. [26]. Copyright 2010 American Chemical Society. (g) Nafion structure with equivalent weight of 1100 (x = 6–7). (h) Schematic of magnetically aligned composite membrane and proton transport in the aligned channels. PWA = phosphotungstic acid, CP4VP = ferrocyanide-coordinated poly(4-vinylpyridine) as electron-donating, proton-conducting, and redox polymer, and PSf = polysulfone as a non-conductive polymer. Reprinted with permission from Liu et al. [30]. Copyright 2019 Springer Nature

In 1998, Ikkala and co-workers demonstrated a switching proton conductivity in PS-*block*-P4VP(MSA)_1.0_(PDP)_1.0_ (PS, polystyrene; P4VP, poly(4-vinly pyridine); MSA, methane sulfonic acid; PDP, pentadecylphenol). Although the proton conductivity was relatively low, they presented structural changes to support change in conductivity using hierarchical order–disorder and order–order transitions (Figure 1(a)) [10]. In 2010, Park and co-workers presented anisotropic proton conduction of poly(styrenesulfonate-*block*-methylbutylene) using domain orientation by pressing, electric-field, and shear-aligned methods (Figure 1(b)) [11]. The pressed sample showed anisotropic proton conduction with σ_||_/σ_⊥_ (the ratio of in-plane conductivity and out-of-plane conductivity) = 75. Chen and co-workers demonstrated anhydrous proton transport in comb polymers with benzotriazole and imidazole [12]. They showed a utility of long decyl chains for organized lamellar and hexagonal nanostructures. In 2015, Matsui and co-workers demonstrated large anisotropic proton conductivity between in-plane and out-of-plane directions using a multilayer thin film with a well-defined lamellar structure by poly(*N*-dodecylacrylamide-*co*-acrylic acid) (Figure 1(c)) [13]. The in-plane and out-of-plane proton conductivity were 5.1 × 10^–2^ and 2.1 × 10^–13^ S cm^−1^, respectively, with σ_||_/σ_⊥_ = 10^11^. This in-plane conductivity is considerably high as a weak acid source of carboxylic acid. It is noteworthy that they assessed the proton conduction mechanisms both theoretically and experimentally using a model of 2D hydrogen-bonding networks in a confined space prepared using Langmuir–Blodgett method (Figure 1(d)) [14–16]. Recently, Winey and co-workers proposed a new but simple polymer design for producing the organizing structure with proton conduction channels [17,18]. They created well-controlled chain folding in sulfonated polyethylene. The linear polyethylene contained sulfonic acid groups pendant precisely to every 21st carbon atom that induced tight chain folds to form the hydrated layers. These reports demonstrate that organized structures are useful not only for high proton conductivity but also for discussing proton conduction mechanisms.

The author and co-workers started research to investigate correlation between structure and proton conductivity for proton-conductive polymer thin films in 2006 (Figure 1(e)) [19,20]. Results showed that proton conductivity is changed by molecular orientation of the polymer according to an interaction between the substrate and the polymer interface [21,22]. In 2008, the first report was made of a study in which the proton conductivity of oligo[(1,2-propanediamine)-*alt*-(oxalic acid)] thin films was improved by the molecular orientation [23–25]. As the oriented structure of other research groups, Tamura and Kawakami presented composite membranes containing uniaxially aligned sulfonated polyimide nanofibers by an electrospinning process (Figure 1(f)) [26]. The composite membranes were prepared using a solvent-cast method to process uniaxially aligned NTDA-BDSA-*r*-APPF nanofibers and exhibiting high proton conductivity, low gas permeability, and good chemical and thermal stabilities (NTDA = 1,4,5,8-naphthalene tetracarboxylic dianhydride, BDSA = 4,4ʹ-diamino-biphenyl 2,2ʹ-disulfonic acid, and APPF = 2,2-bis[4-(4-aminophenoxy)phenyl]-hexafluoropropane). Nafion is the most widely investigated as a proton-conductive polymer (Figure 1(g)) [27,28]. In 2010, Elabd and co-workers demonstrated highly proton-conductive Nafion nanofibers [29]. The proton conductivity of single high-purity Nafion nanofiber was found to be 1.5 S cm^−1^: an order of magnitude higher than that of bulk Nafion membranes. Guiver and co-workers demonstrated magnetic-assisted proton-conductive membranes (Figure 1(h)) [30]. These enhancements in proton conductivity are regarded as based on molecularly oriented structures. In recent years, the molecular design of self-organized structures for high proton conduction has become increasingly important [21,31]. In this review, several current topics are introduced, particularly focusing on highly proton-conducting polymer thin films based on organized, molecularly oriented structure. Though the author does not cover anhydrous proton-conductive films in this review, several recent literature would be introduced at the end of Section 3.2 for readers.

## 2. Highly proton-conductive polymer thin films with molecularly oriented structure

### 2.1. Perfluorinated sulfonic-acid thin films

For the last 10 years, the study of ‘thin’ Nafion ionomers has attracted researchers because ionomer is necessary for fuel cell reactions [21,28,32]. Thin ionomers serve to transport protons from the proton-conductive membrane to the electrochemical catalyst in fuel cells. Protons are transported through the thick membrane but along the thin ionomer at the interface in catalyst layers. Therefore, the proton conductivity in the in-plane direction becomes important for thin ionomers. Since Siroma and co-workers reported declining in-plane proton conductivity with decreasing thickness of a Nafion thin film [33], the relation between the interfacial structure of perfluorinated sulfonic acid ionomer and proton transport properties has been discussed to an increasing degree. The author would like to introduce our progress in this area, including discussion of other related works from 2017 [21].

**Figure 2.**
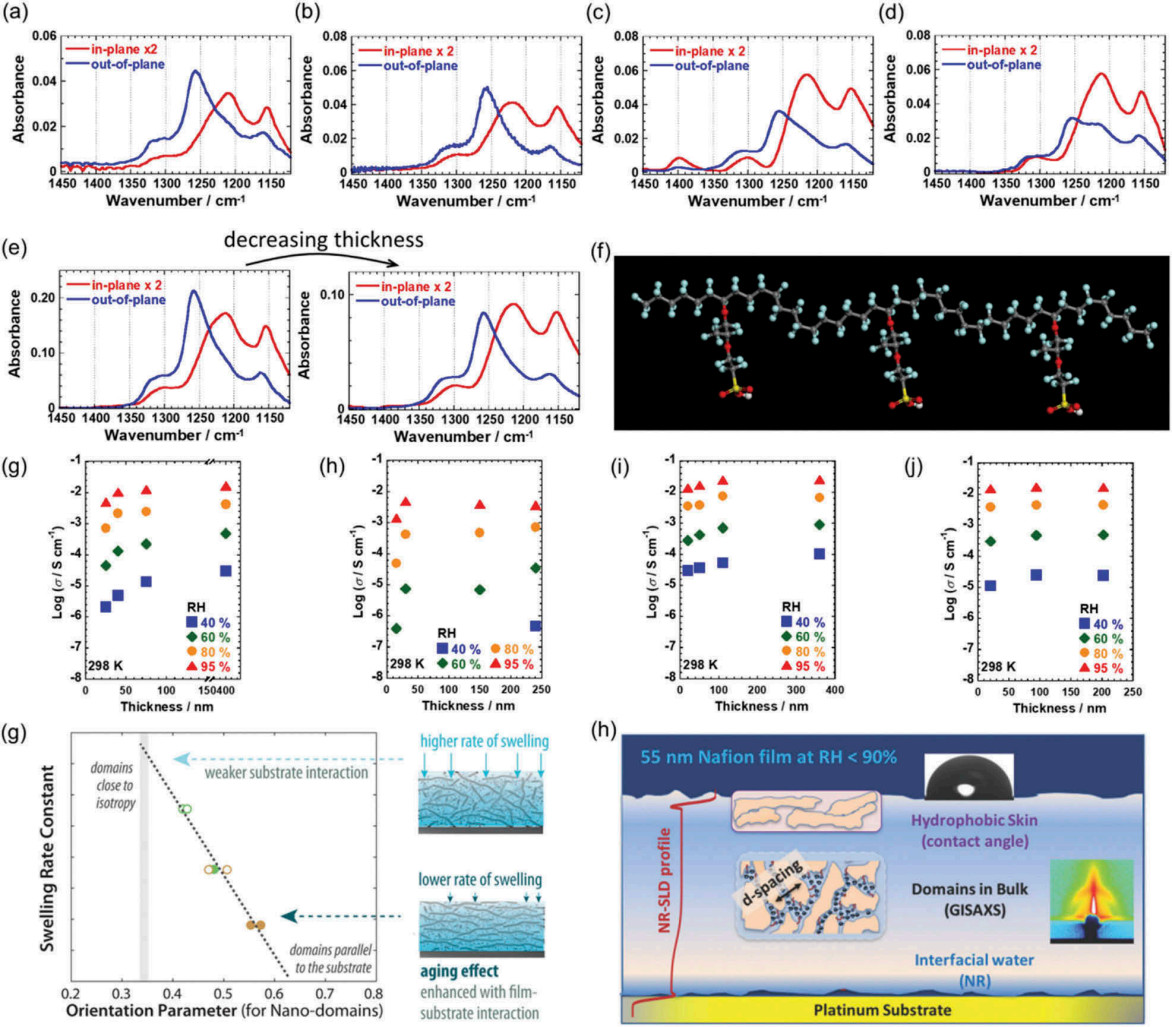
(a) pMAIR spectra of Nafion thin film on the SiO_x_ surface. (b) pMAIR spectra of 30-nm-thick Nafion thin film on the MgO surface. (c) pMAIR spectra of 35-nm-thick Nafion thin film on the sputtered Pt surface. (d) pMAIR spectra of 20-nm-thick Nafion thin film on the sputtered Au surface. (e) Thickness dependence of pMAIR spectra of 170-nm-thick and 80-nm-thick Nafion thin films on the sputtered Pt surface. (f) Structure of Nafion calculated using density functional theory. (g) Thickness dependence of proton conductivity for Nafion thin film on the quartz surface. (h) Thickness dependence of proton conductivity for Nafion thin film on the MgO surface. (i) Thickness dependence of proton conductivity for Nafion thin film on the sputtered Pt surface. (j) Thickness dependence of proton conductivity for Nafion thin film on the sputtered Au surface. (g) Orientation parameter parallel to the substrate surface vs. swelling rate constant. Reprinted with permission from Tesfaye et al. [71]. Copyright 2019 American Chemical Society. (h) Schematic of hydration-dependent microscopic hydrophilic domains and macroscopic expansion of 55 nm-thick Nafion film on a Pt surface. Reprinted with permission from Shrivastava et al. [72]. Copyright 2020. The royal society of chemistry

Our group reported the highly oriented structure of Nafion thin films on SiO_x_ [34], MgO [35,36], sputtered Pt [37], and sputtered Au [36] surfaces by infrared *p*-polarized multiple-angle incidence resolution spectrometry (pMAIRS), which was developed by Hasegawa and co-workers [38–40], as presented in Figure 2(a–d). IR pMAIRS offers the molecular orientation for each functional group to various functional materials such as derivatives of polythiophene [41,42], porphyrin [43], pentacene [44], fullerene [45], naphthalene diimide [46], phthalimide [47], azulene [48], metal-oxide nanowire [49], polymer brushes [50], and polyimide [51]. Because this spectroscopic method is an infrared spectroscopic method, it is useful because it is applicable to non-crystalline materials. In combination with X-ray scattering technique, it is useful to discuss the structure of crystalline and amorphous parts in other materials [43,44,47,48,52–54]. It also makes it possible to discuss the substrate dependence in greater detail [25,36,37] or casting solvent dependence [43,54] of the interfacial structure.

Returning to Nafion thin films, bands at 1150, 1210–1240, and 1300 cm^−1^ in Figure 2(a–d) can be assigned to *ν_as_* (CF_2_), the mixture of *ν_as_* (CF_2_) and *ν_as_* (SO_3_^−^), and *ν* (C-C) [55]. The characteristic band at 1260 cm^−1^ was observed only in the out-of-plane spectrum. This attribution of the absorption band remains unsolved. Some attributions were suggested as *ν_as_*(CF_3_) + δ_s_(COC) [56], *ν_as_*(CF_2_) [57], *ν* (CF_2_) [58], *ν_as_*(CF_3_) [59], and – SO_3_^−^ [60,61] vibration modes. Karan and co-workers detected a thickness-dependent band peaking at 1223–1259 cm^−1^ in Nafion films by attenuated total reflection Fourier-transform infrared (ATR-FTIR) spectroscopy [62]. However, our band position was thickness-independent for thicknesses of 20–210 nm. Our observed band at 1260 cm^−1^ by pMAIRS can be regarded as having different origin with the reported thickness-dependent peak by ATR measurements [57,62]. The oriented structure of Nafion thin films depends on the thickness of sputtered Pt and Au surfaces [36,37], although this large structural change was not observed in Nafion thin films on SiO_x_ and MgO surfaces. The band at 1210–1240 cm^−1^ in the in-plane spectrum on sputtered Pt and Au surfaces was enhanced with decreasing thickness compared to the band at 1260 cm^−1^ in the out-of-plane spectrum (Figure 2(e)). Considering the structure of Nafion calculated using density functional theory as presented in Figure 2(f), this band intensity is derived mainly from the *ν_as_* (CF_2_) modes of the main chain. Yagi and co-workers demonstrated that the SO_3_^−^ groups were oriented to the Pt surface in the ca. 5 nm region of the Nafion/Pt interface characterized by vibrational sum frequency generation spectroscopy [63]. Our results indicate that main chains on sputtered Pt and Au surfaces were oriented to the in-plane direction to the substrate surface and that the main chain orientation was enhanced with decreasing thickness. This discussion of main-chain orientation can be supported by the work of another research group [64].

Figure 2(g–j) presents the film thickness dependence of the in-plane proton conductivity on quartz [37], MgO [35,36], sputtered Pt [37], and sputtered Au [36] surfaces. All proton conductivities showed lower values than those of a commercially available Nafion membrane [35,65,66]. This lower proton conductivity is a widely reported result [33,67–70]. The decreasing trend in proton conductivity was found to depend on the substrate surface. On quartz and MgO substrates, the proton conductivity decreased concomitantly with decreasing thickness and conductivity drop occurring at around the 15–40 nm thick. On the sputtered Pt surface, the conductivity decreased slightly with decreasing thickness. Furthermore, on the sputtered Au surface, the conductivity was almost a constant value of 1.5–1.7 × 10^−2^ S cm^−1^ for thicknesses of 20–200 nm at 95% relative humidity (RH) and 298 K [36]. Results indicate that the metal-deposited surfaces suppressed the conductivity drop in thinner films. Our group speculates that the fundamental origin of the suppression of conductivity drop might derive from the highly oriented main chains, as described in the preceding paragraph.

Tesfaye and co-workers reported the nanodomain orientation and swelling kinetics by hygrothermal aging in perfluorosulfonic acid thin films of two types [71]. They described that the higher the orientation parallel to the substrate surface becomes, the slower water is transported normal to the surface (Figure 2(g)). Shrivastava and co-workers demonstrated evolution of hydration-dependent microscopic hydrophilic domains and macroscopic expansion of 55 nm-thick Nafion film on a Pt surface (Figure 2(h)) [72]. Cross-correlation among the film macro-expansion from ellipsometry, micro-expansion from grazing incidence small angle X-ray scattering (GISAXS), and the water distribution from neutron reflectometry showed randomly and spatially non-uniform distribution of water domains. Discussion involving proton conductivity as a macroscopic factor and domain size and swelling as microscopic factors revealed that a tortuosity of proton conduction pathways, which has an inverse relation with proton conductivity [73], was found to be inversely proportional to the domain expansion. These reports also support the oriented structure of Nafion thin films.

The current direction of studies of perfluorinated sulfonic acid ionomers includes not only studies of other perfluorinated ionomers but also studies of the effects of environmental conditioning and different substrate surfaces [32,74–80]. Although not addressed in this review, oxygen transport loss to the ionomer thin films is an important objective of continuing study [72,81–83].

### 2.2. Synthetic polypeptide thin film

**Figure 3.**
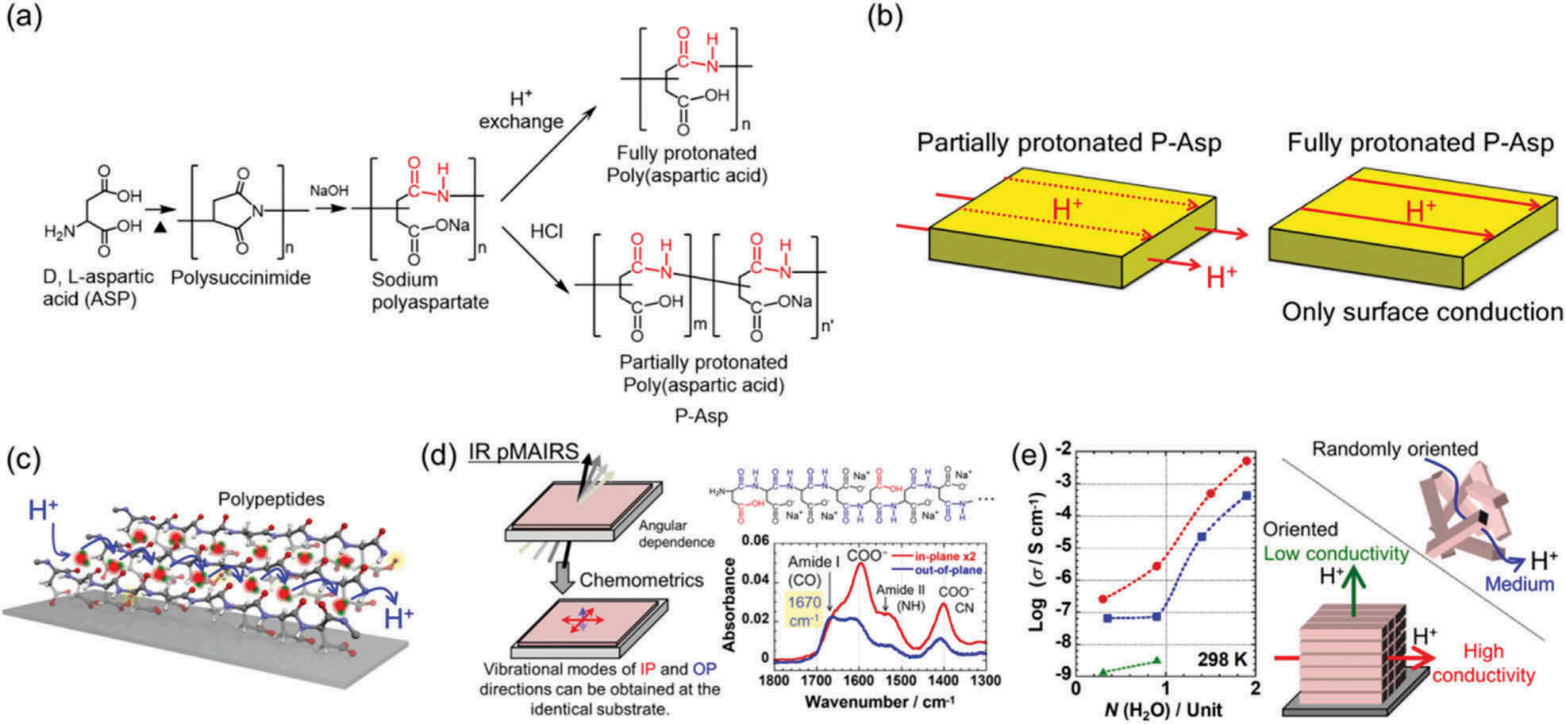
(a) Synthesis scheme of fully and partially protonated poly(aspartic acid). Reprinted with permission from Nagao [21]. Copyright 2017 American Chemical Society. (b) Schematic of surface proton conduction internal proton conduction respectively in the fully protonated poly(aspartic acid) thin film and partially protonated thin film. Adapted with permission from Nagao et al. [86]. Copyright 2014 Elsevier. (c) Proposed structure of the partially protonated thin film with nonperiodic α-sheet-like structure. Pink atoms surrounded by yellow atoms represent proton carriers of carboxylic acid groups: C, gray; N, blue; O, red; and H, white and green. In-plane direction is parallel to the substrate surface. Blue arrow represents enhanced proton conduction in the in-plane direction through hydrogen bond networks. (d) Schematic of IR pMAIRS technique and pMAIR spectra. In-plane direction is parallel to the substrate surface. In-plane and out-of-plane spectra show different shapes. Therefore, some functional groups have oriented structure. (e) Anisotropic proton conductivity and schematic of proton conduction in oriented and randomly oriented samples. N denotes the number of water molecules per polymer unit

Amino acid-based polymers take several hierarchical structures such as α-helix or β-sheet via hydrogen bond networks between amide groups. Our group investigated synthetic poly(aspartic acid) thin films to elucidate the relation between the oriented structure and anisotropic proton conduction. Poly(aspartic acid) was polymerized synthetically from a monomer of D, L-aspartic acid through polysuccinimide (Figure 3(a)) [84,85]. This synthetic poly(aspartic acid) had an unusual proton transport property. The thin films of fully protonated poly(aspartic acid) did not conduct proton inside of the thin film; only surface proton conduction was observed (Figure 3(b)) [86]. However, thin films of partially protonated poly(aspartic acid)/sodium polyaspartate (P-Asp) showed proton conduction inside of the thin film and exhibited anisotropic proton conduction between in-plane and out-of-plane directions of the thin film [87]. The mobile carrier was determined as a proton by checking H/D isotope effects and the open circuit potential [88]. The P-Asp thin films showed a molecularly oriented structure of amide groups, as portrayed in Figure 3(c). This oriented structure was confirmed using IR pMAIRS (Figure 3(d)). The absorption band of the C = O amide group as amide I band at 1670 cm^−1^ can be assigned as a nonperiodic α-sheet-like structure [89]. Table 1 presents the absorption band region and possible assignments. Because the signal intensities of the amide I band at 1670 cm^–1^ for the in-plane and out-of-plane spectra were comparable, the average transition moment vector of the amide groups is determined to be directed ca. 45 degrees from the substrate surface. The angle can be estimated according to the following Equation (1),

(1) $$\varphi =\tan^{-1}\sqrt{\frac{2I_{IP}}{I_{OP}}}$$

**Table 1.** Possible assignments of structures and absorption band regions of amide I [89]

Structure	Amide I/cm^−1^
α-helix	1640–1660
β-sheet	1620–1640
Random coil	1640–1660
Others (turn, bulge, loop, α-sheet, etc.)	>1660

where *I*_IP_ and *I*_OP_ are the IP and OP peak absorbance of the amide I band and *φ* is the orientation angle from the surface normal. The symmetric and anti-symmetric stretching bands of the COO^–^ group at 1400 and 1600 cm^−1^ gave a stronger signal in the in-plane spectrum than twice the intensity of the out-of-plane spectrum, which indicates that the O–C–O plane of the COO^–^ group at the side chains lies parallel to the substrate plane. Considering those results together, our group proposed the non-periodic α-sheet-like model with a main chain-oriented structure, as portrayed in Figure 3(c). This model does not necessarily mean that the thin film is composed exclusively of α-sheet layers. This unusual oriented structure might be derived from the flat surface of the substrate and interactions of hydrogen bonds through a polymer–polymer and polymer–substrate surface. From this structural model, anisotropic proton conductivity was expected between the in-plane and out-of-plane directions to the film. Figure 3(e) shows the amount of water dependence of the proton conductivity for the 60-nm-thick film. The in-plane proton conductivity (2.7 × 10^−6^ S cm^−1^) was much higher than the out-of-plane conductivity (3.4 × 10^−9^ S cm^−1^) at 50% RH and 298 K [87]. The randomly oriented pelletized sample exhibited medium conductivity between in-plane and out-of-plane conductivity. Results reveal that the proton conductivity is enhanced to the in-plane direction through the P-Asp oriented structure. We also examined the effects of centrifugal force during spin-coating to prepare thin films. Results indicate that no difference of proton conductivity exists between the radial direction and the direction perpendicular to it in P-Asp thin films [87]. As mentioned at the beginning of Section 2.1, the proton conductivity in the in-plane direction is important in catalyst layers because protons are transported through the membrane (through-plane) but along the interface on electrochemical catalysts and porous carbons in catalyst layers (in-plane).

## 3. Highly proton-conductive polymer thin films with organized structure

### 3.1. Alkyl sulfonated polyimide thin films

**Figure 4.**
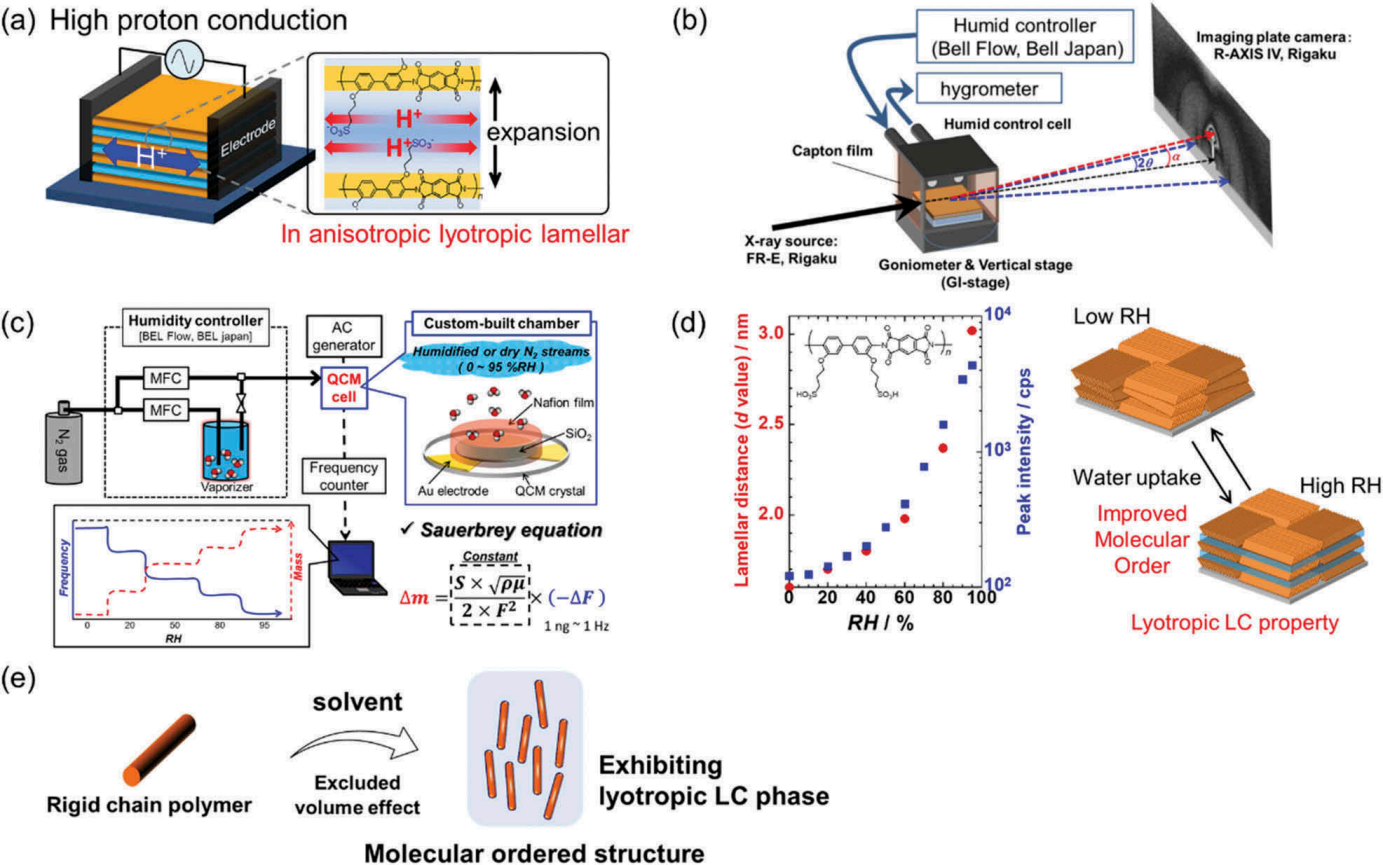
(a) Schematic of organized lamellar structure with high in-plane proton conductivity of 10^−1^ S cm^−1^ lamellar expansion dependent on the amount of water uptake. (b) Schematic of humidity controlled in situ grazing incidence small angle X-ray scattering (GISAXS). (c) Schematic of humidity controlled in situ quartz crystal microbalance (QCM). MFC means a mass flow controller. (d) Relative humidity (RH) dependence of lamellar distance and peak intensity. Schematic of reversible structural change of organized lamellar structure by water adsorption/desorption process. The degree of molecular order also improved with the water uptake. (e) Schematic of lyotropic LC property using rigid chain polymers for molecular order

Sulfonated polyimides (SPIs) have been reported since 1997 as alternative proton-conductive membranes for fuel cells because of their high chemical and thermal stability [90–95]. The author, in addition to Nagano and co-workers found that the thin film forms of alkyl sulfonated polyimides (ASPIs) exhibited an organized lamellar structure parallel to the substrate surface and high in-plane proton conductivity of 10^−1^ S cm^−1^ at 298 K (Figure 4(a)) [96]. This lamellar expansion depends on the amount of water uptake. Results of humidity-controlled in situ GISAXS (Figure 4(b)) and humidity-controlled in situ quartz crystal microbalance (QCM, Figure 4(c)) revealed that the lamellar distance linearly depends on the number of water molecules. Also, the degree of molecular order improved with the water uptake (Figure 4(d)). This structural change occurred reversibly with the water amount. Formation of the organized lamellar structure and improvement of the molecular order were derived from a lyotropic liquid crystalline (LC) property. In their pioneering study, Wegner and co-workers reported on lyotropic LC property using rigid main chains [97]. The origin of the lyotropic LC property originates from the fact that the main chains are aligned in one direction because of the excluded volume effect of the main chain in the solvent (Figure 4(e)) [98]. ASPIs are a promising candidate because they have not only high solubility because of the high polarity of the sulfonic acid groups at the side chains but also rigid and rod-shaped main chain. The following paragraphs describe our recent investigation of correlation between the structures of ASPI thin films and proton conduction. Our earlier works related to ASPI thin films are summarized in other reports of the literature [21,22].

**Figure 5.**
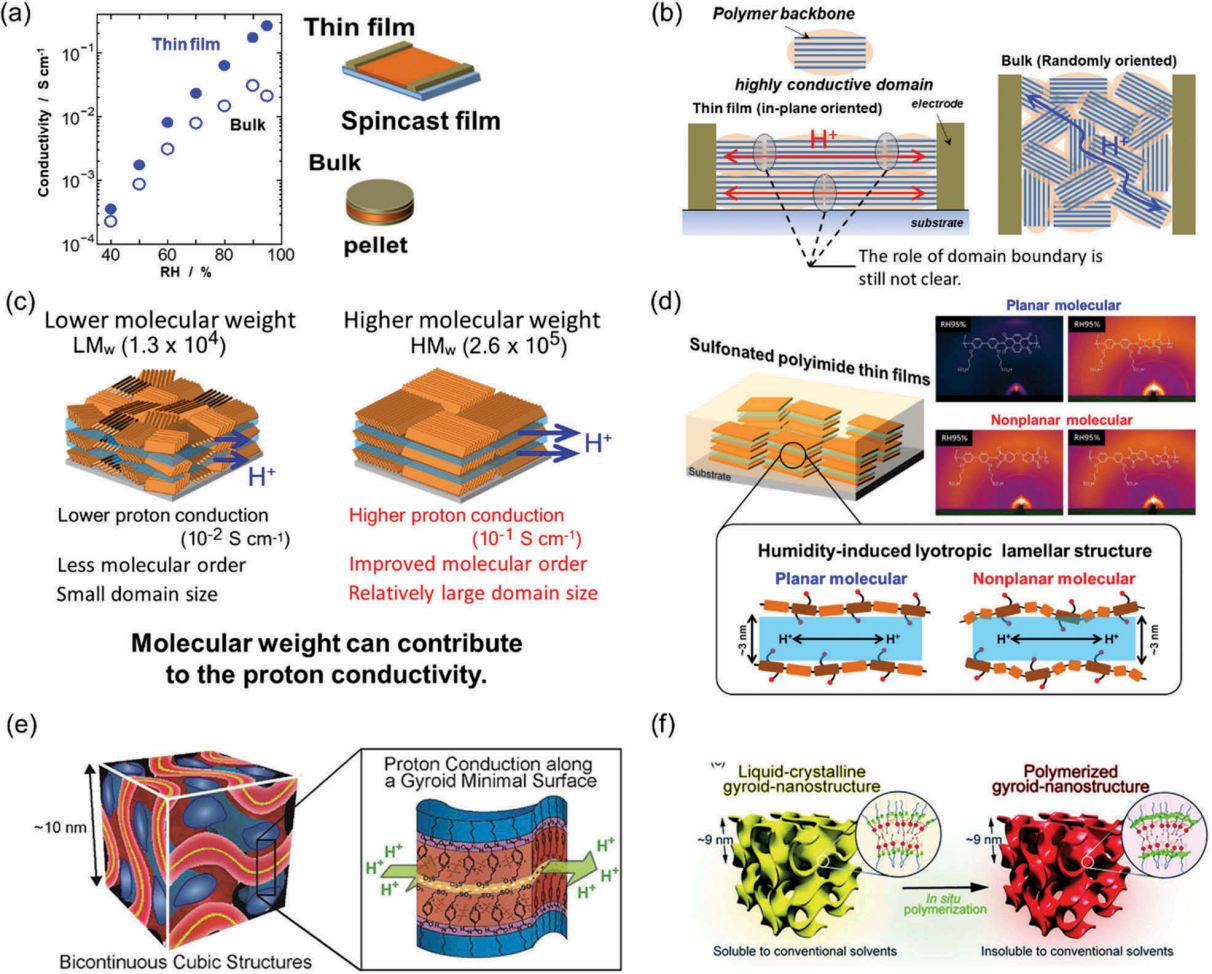
(a) Relative humidity (RH) dependence of proton conductivity of ASPI between the thin film and pelletized sample. (b) Schematic of in-plane oriented lamellar domain of thin film and randomly oriented domain for the pelletized sample. In thin film, the flat substrate surface drove the in-plane orientation. (c) Schematic of molecular weight dependence of proton conductivity and molecular order. ASPI thin film with high molecular weight exhibited higher molecular order and higher proton conductivity than that with low molecular weight. (d) Schematic of influence of rigidness and planarity of the main chain to the organized structure in ASPI thin films Proton conductivity with planar backbone was higher than that with bent backbone [101]. Reprinted with permission from Ono et al. [101]. Copyright 2018 American Chemical Society. (e) Schematic of bicontinuous cubic LC structure and proton conduction pathway along the gyroid minimal surface. Reprinted with permission from Ichikawa et al. [112]. Copyright 2012 American Chemical Society. (f) From a molecular-based LC gyroid-nanostructure to insoluble polymerized gyroid-nanostructure. Reprinted with permission from Kobayashi et al. [108]. Copyright 2019. The royal society of chemistry

Molecular orientation is a useful structural parameter to enhance proton conductivity [21]. Our studies demonstrated that the in-plane oriented lamellar structure of ASPI thin films exhibited much higher proton conductivity than that of pelletized samples (Figure 5(a)) [96,99]. In pelletized samples made from powder, the domains of lamellar structure were formed, but these organized domains were oriented randomly (Figure 5(b)). In the thin films, the flat substrate surface was able to drive the formation of lamellar structure in the in-plane direction to the substrate surface. This result illustrates the importance of molecular orientation for proton conduction.

Molecular weight is another useful parameter to enhance ASPI thin film proton conductivity [100]. In general, the molecular weight of proton-conductive polymers contributes to mechanical strength, but it does not contribute to proton conductivity. In ASPI thin films composed of pyromellitic dianhydride and 3,3ʹ-bis(3-sulfopropoxy)benzidine, the molecular weight is related strongly with the degree of molecular order. Results of GISAXS revealed that the ASPI thin film with high molecular weight (2.6 × 10^5^) exhibited higher molecular order and higher proton conductivity than that with low molecular weight (1.3 × 10^4^), as depicted in Figure 5(c). The proton conductivities of ASPI thin films with high and low molecular weight, respectively, showed 2.6 × 10^−1^ and 1.0 × 10^−2^ S cm^−1^ at 298 K and 95% RH. Larger ordered domains in the high-molecular-weight ASPI thin film affect proton-conductive property because fewer LC domain boundaries exist than in the low-molecular-weight ASPI thin film. Elucidating domain size and domain boundary roles for highly proton-conductive polymers is still an attractive issue for clarifying proton conduction mechanisms.

Recently, Ono and co-workers discussed the influence of rigidity and planarity of the main chain structure in ASPI thin films (Figure 5(d)) [101]. Earlier studies showed interchain packing for the lamellar structure as suppressed by steric effects of nonplanar and bent backbones of polyimides without sulfonic acid group [102]. Four ASPIs with sulfonic acid groups were synthesized to elucidate the influence of the planarity of the ASPI backbone using planar and bent backbones. Results of GISAXS revealed that both planar and bent ASPI thin films exhibited a humidity-induced lyotropic lamellar structure. Both enhanced the molecular order with lamellar structure expansion up to 2.9–3.1 nm to the out-of-plane direction by water uptake. The structure of amphiphilic polyimides with alkyl sulfonated side chains plays a role in forming the organized lamellar structure by lyotropic LC property. It is particularly interesting that the planar ASPI main chains exhibited a smectic phase and that vent main chains showed a nematic phase. Results demonstrated that the backbone planarity affects the arrangement of interchain packing in the organized lamellar structure. In fact, the in-plane proton conductivity with planar backbones exhibited a higher value (10^−1^ S cm^−1^) than that exhibited by bent backbones (10^−2^ S cm^−1^). The higher degree of molecular order enhances proton conductivity.

Takakura and co-workers demonstrated the influence of a semialiphatic 1,2,4,5-cyclohexanetetracarboxylic dianhydride backbone between molecular order and proton conductivity [103]. In an earlier study, Ando and co-workers studied the molecular aggregation structure of both fully aromatic and semialiphatic polyimides without a sulfonated alkyl side chain [102]. They described that steric effects of the polyimide backbone without alkyl sulfonated side chains have influenced the interchain packing structure. Our work showed that the molecular order was weakened by the introduction of semialiphatic backbone because of suppressed (π-stack) interchain packing in the lyotropic LC structure. Scattering corresponding to the lamellar structure was isotropic and weak. However, the degree of molecular order and in-plane orientation improved with increasing molecular weight. By this structural change, the proton conductivity also improved from 3.0 × 10^−2^ S cm^−1^ to 1.5 × 10^−1^ S cm^−1^.

### 3.2. Other recent organized films with proton-conductive channels

Several groups have also recently reported proton conduction using an organized structure with thermotropic and lyotropic LC properties [104–110]. Ohno and co-workers reported anisotropic proton conduction by a self-assembled lyotropic columnar structure using phosphonium-type zwitterions and bis(trifluoromethanesulfonyl)imide [111]. Zwitterions, in which both cation and anion are bonded covalently, are anticipated for use for selective ion transport. Ichikawa and co-workers demonstrated high proton conduction by 3D continuous water nanochannels as a gyroid structure using amphiphilic zwitterions and bis(trifluoromethanesulfonyl)imide [112]. This configuration exhibited a bicontinuous cubic LC structure (Figure 5(e)). They developed this system from a molecular-based LC gyroid nanostructure to a polymerized gyroid nanostructure, which is insoluble by conventional solvents (Figure 5f) [108]. The film showed high ionic conductivity of ca. 10^−1^ S cm^−1^ at room temperature.

Yabu and co-workers demonstrated proton conduction channels along the lamellar organized structure using block copolymer thin films composed of poly(vinyl catechol) and polystyrene [113]. The proton conductivity was increased ten-fold by the addition of silver nanoparticles into the proton conduction channels filled with catechol moieties. He and his co-workers also reported proton conduction channels using mussel-inspired catechol-containing triblock copolymers composed of poly(methyl methacrylate), poly(vinyl catechol), and polystyrene [114]. The synthesized triblock copolymer formed a cylindrical organized structure in which poly(vinyl catechol) domains are located on the cylinder surface. This organized structure functioned as a template for silver nanoparticle arrays and proton-conductive channels.

The author does not cover anhydrous proton-conductive films in this review, however, several recent publications are referred to as anhydrous proton-conducting films for designing organized structures and molecularly oriented structures [115–117]. Park and co-workers reported lamellar organized structures with high-dielectric constant crystalline proton-conductive channels using single-ion conducting block copolymers and twitter ion additives [115]. They discussed synergistic dipole alignments for improving proton transport properties. The optimized sample exhibited a high proton diffusion coefficient of 2.4 × 10^−10^ m^2^ s^−1^, which was determined by a pulsed field gradient method of ^1^H nuclear magnetic resonance, under anhydrous condition at 90°C.

Organized structures can not only form highly proton conducting channels: they might also control anisotropic proton conduction through structural control. Controlling proton conductivity is not easily accomplished with amorphous polymers because they have less long-range molecular order. The organized structure is expected to play an important role in controlling proton conduction in the near future.

## 4. Summary and outlook

The author summarized recent progress on recent highly proton-conductive polymer thin films with an organized structure and a molecularly oriented structure. The organized structure and molecularly oriented structure are anticipated as promising approaches not only to make highly proton-conductive channels but also to elucidate the mechanisms of high proton conduction. Development of polymer design and induction techniques by external fields for obtaining organized structure will continue to be important. As one avenue of study using the advantage of organized structure in the near future, control of anisotropic proton conduction might be achieved by application of external fields. For such studies, polymer design using thermotropic and lyotropic LC properties and/or hydrogen bond networks between polymer backbones will become increasingly important.
